# Reassessing onco-exceptionalism: equity and resource allocation in immunotherapeutic cancer treatments

**DOI:** 10.1136/jme-2025-110739

**Published:** 2025-04-21

**Authors:** Hamideh Frühwein, Nikolai Münch, Norbert W Paul

**Affiliations:** 1 Institute for the History, Philosophy and Ethics of Medicine, University Medical Center of the Johannes Gutenberg University Mainz, Mainz, Germany

**Keywords:** Ethics- Medical, Health Care Economics and Organizations, Policy

## Abstract

Given that only a small fraction of patients with cancer exhibits specific markers making them eligible for effective targeted therapies, this paper investigates the justification of treating cancer differently in terms of resource allocation when it comes to the application of novel precise therapies—the so-called onco-exceptionalism. Specifically, it assesses whether the reimbursement of expensive drugs is equitable. To do so, we first contextualise healthcare resource allocation concerning immunotherapeutic treatments for cancer, then explore arguments for and against onco-exceptionalism and finally conclude by advocating for a proactive health approach.

## Introduction

 The allocation of healthcare resources, particularly in the realm of cancer treatment, poses a significant challenge for policymakers and healthcare providers. Current policy discussions stress the need for more research on the intersection of coverage laws and oncology care.^1^

Given the finite financial resources in healthcare, the escalating costs of novel cancer treatments inevitably prompt distributional concerns. In addressing these allocation dilemmas, policymakers nowadays heavily rely on cost-effectiveness analysis (CEA). CEA typically involves assessing quality-adjusted life years (QALYs) alongside the incremental cost-effectiveness ratio. This method evaluates the financial impact and health benefits of new interventions compared with existing options, guided by society’s willingness to pay (WTP). The WTP threshold is an economic concept used in health economics to determine whether an intervention provides sufficient health benefits to justify its cost. It represents the maximum amount a healthcare system or society is willing to pay for a unit of health benefit (eg, a QALY). If the cost of an intervention is below this threshold, it is considered cost-effective; if it exceeds the threshold, it may not be considered cost-effective.^2^

While CEAs are designed to maximise overall health gains (and therefore the overall effectiveness of healthcare spending), the distributional effects (ie, who actually benefits from these health interventions and how resources are distributed across different groups in society) are often overlooked. This omission of distributional concerns does present an ethical challenge, as CEA might unintentionally lead to health inequities. For example, when health insurers, government health bodies and health systems use CEA to decide which treatments to reimburse, certain groups may be excluded. This may be seen as a concomitant bias as illustrated by Loree *et al*^3^ and Pierce,^4^ with an under-representation of racial minorities, and by Unger *et al*^5^ for patients in low-income communities in certain studies. The advent of immunotherapies and combination therapies in oncology has further complicated this landscape due to a higher level of segmentation of patient groups accompanied by substantially rising costs, a trend that is projected to persist in the foreseeable future.^6^ This escalation is furthermore attributed to the introduction of new medications, broadening of treatment indications and prolonged therapy duration resulting from desirable enhanced patient outcomes.^7^ Against this context, we would like to pose questions regarding a particular attention given to cancer, onco-exceptionalism, and evaluate the pros and cons of such exceptionalism from the perspective of justice in the allocation of healthcare resources.

According to Fu *et al*,^8^ onco-exceptionalism refers to the perception and treatment of cancer as distinct from other medical conditions, often leading to unique considerations and approaches. This differentiated treatment is evident not only in specific reimbursement decisions but also in four primary areas: development and funding,^9^ approval processes for new drugs,^10 11^ cancer drug pricing^8^ and coverage.^12^

Cancer research funding is disproportionately high (in absolute figures and relative to population’s disease burden^13^) with cancer accounting for the largest share of new drug approvals.^9^ Cancer drugs often benefit from special regulatory pathways, like accelerated approval, enabling quicker access but sometimes based on less robust evidence.^8 14^ Despite rising costs, in the USA, for example, studies show no consistent correlation between price and clinical benefit.^8^ Some reimbursement policies also reflect onco-exceptionalism, which enables higher cost-per-QALY thresholds for cancer drugs^8^ as is the case for the UK cancer drug fund.^12^ This phenomenon raises questions about the fairness and efficiency of such policies, especially in light of the financial burden placed on healthcare systems.^15^ Therefore, the following two questions will be addressed:

1. What are the arguments in support of and against such an onco-exceptionalism?
2. Based on the reflections on precision oncology and different ways to adopt reimbursement decisions, can an onco-exceptionalism be justified and if so, what kind?

## Arguments *for* and *against* increasing the threshold

Advocates for raising the WTP threshold in immunotherapeutic treatments for cancer present several ethical justifications, among which are the severity of illness, the ‘rule of rescue’, the distinction between individual health gains and population health metrics, and equity concerns, all emphasising the moral obligation to prioritise life-saving treatments for patients with advanced cancer and limited options. Opponents of onco-exceptionalism challenge these arguments. To focus our analysis, in the following subsections, we will concentrate on two key issues as examples.

### Severity of illness

One of these key arguments is that health conditions that are judged to be more severe should be given priority and more resources from an ethical perspective, even if this means a loss in aggregate health benefits and therefore lower total cost effectiveness. Such a priority to the worse off in the sense of severity of disease is supported by different ethical positions—be it Kantian inspired deontological views, egalitarian concerns or prioritarian views—although with different ethical arguments.^16 17^ Also, the understanding of what severity means varies, both philosophically and in public opinion.^16 18^ Public preference studies consistently show a strong tendency to prioritise interventions for severe health conditions, even when these interventions are less effective than alternatives.^17^ In this context, severity is typically understood as a condition that is serious, progressive, and likely to result in premature death.^12^ Given the missing consent on severity and its role in healthcare resource allocation, Brock^19^ raises three essential questions regarding the prioritisation of the worst-off: why prioritise the worse-off, who qualifies as the worse-off, and how much priority should they receive? He suggests that it may be justifiable to limit prioritisation to individuals with poorer health. However, he also highlights other considerations, including criteria for defining who is worse-off—whether by overall health status or the severity of the current condition—and whether to consider present health, lifetime health or future health in decision-making. He also discusses fairness in terms of equity in lifetime health, which would mean that those furthest from reaching a full lifespan should have a stronger claim for additional years of life.

Notwithstanding these open questions, the focus on severe, life-threatening conditions reflects a widely accepted moral intuition that life-saving interventions should take precedence, which could be justified from different ethical positions. However, this approach risks oversimplifying healthcare prioritisation by overly focusing on severity while mostly neglecting treatment effectiveness, cost-efficiency and long-term societal health. Although public preference studies indicate a preference for prioritising severe health conditions, trade-off studies by Norheim *et al*^17^ reveal that individuals place more weight on overall health than on the seriousness of a specific condition. By prioritising acute conditions regardless of treatment effectiveness, resources may be misallocated, particularly when compared with more treatable, less severe conditions.

This critique is exemplified in the concept of onco-exceptionalism.^20^ First, the severity argument is also indifferent to the effectiveness of the treatment and overlooks the opportunity costs.^12^ For example, the overpricing of cancer drugs with limited evidence of clinical benefit is evident in the case of imatinib in the USA, where high costs persist despite the drug’s modest survival benefit,^11 21^ and the UK’s Cancer Drug Fund, which has faced criticism for funding high-cost drugs with low levels of evidence of their effectiveness.^12 22 23^ As a consequence, ineffective treatments for severe cases would command a higher priority for funding in a benefits package than highly effective interventions for less severe conditions.^12^ For the same reason, providing relatively cost-ineffective treatments would deny care to others whose conditions were judged to be insufficiently severe, despite the availability of highly effective and relatively cheap interventions.

Furthermore, a healthcare system focused only on the worse-off may fail to recognise the importance of addressing preventable health inequalities. Preventive healthcare, however, could offer both individual and societal benefits. From an individual perspective, prevention helps avoid suffering, while from a societal perspective, it can delay or prevent conditions requiring expensive treatments. By targeting preventive care, resources can be redistributed to those at risk but not yet ill, ensuring healthcare systems remain responsive to the health needs of entire populations, not just the most vulnerable. While the cost-effectiveness of prevention and early detection is not universally proven, there is evidence supporting their effectiveness in many contexts, particularly when targeted at high-risk populations.^24^,^27^

### High costs

The argument that cancer research and development (R&D) is inherently more innovative than other clinical R&D, thus justifying its prioritisation at the potential expense of other healthcare needs, is largely driven by onco-exceptionalism and competition in scientific, clinical and economic fields. The National Institute for Health and Care Excellence (NICE) previously adopted a higher cost-per-QALY threshold for end-of-life drugs, creating a dual threshold system for cancer therapies and other medical interventions. This approach has been criticised for undermining NICE’s decision-making framework and weakening incentives for pharmaceutical companies to align pricing with health benefits.^28^

Our position on prioritising cancer R&D is twofold. First, as was mentioned earlier, while cancer represents a major public health issue, it is not the only area of rapid innovation. Fields such as autoimmunity and inflammation are also advancing, and prioritising cancer at the expense of these areas could hinder progress in other important healthcare domains. Second, the high cost of cancer treatments often does not correspond with their clinical benefits. Studies by Vokinger *et al*^11^ and Furlow^29^ show that US cancer drug prices are exorbitant without expected proportional improvements in patient outcomes. The value of any medication depends on its overall clinical benefits, that is improved survival and quality of life (desirability), and its cost (affordability). After examining medication prices in England, Germany, France and Switzerland, all of which employ robust health technology assessment processes and engage in price negotiation, Vokinger and colleagues^11^ concluded that this assumption is flawed. Their study encompassed 65 drugs, of which 47 (72%) were designated for solid tumours and 18 (28%) for haematological malignancies. In the USA, the median monthly cost of drug treatment was 2.31 times higher (with an IQR of 1.79–3.17) compared with the assessed European countries. Notably, there were no significant correlations between the monthly treatment costs for solid tumours and the clinical benefits observed across all examined countries. US cancer drug prices do not reflect increased benefits to patients.^29^

Additionally, concerns about the regulatory approval process for cancer drugs, particularly in the USA, suggest that less rigorous evaluation may undermine claims for prioritising cancer R&D over other areas with stricter regulatory scrutiny.^8 30^ Plutynski^31^ illustrates how precision oncology may not always deliver significant real-world benefits. For example, the TAILORx study demonstrated that many women with early-stage breast cancer do not benefit from adjuvant chemotherapy, even though the Oncotype DX test, despite its high cost, provided redundant information compared with existing clinical methods. These findings suggest that precision medicine often presents an idealised vision of future possibilities that may not align with present-day realities. Maynard and Bloor^28^ also argue that political motivations, such as the British Conservative Party’s commitment to funding cancer drugs, risk depriving other patients of necessary care and undermining evidence-based policy-making. Selective funding for cancer, while politically appealing, conflicts with the ethical goal of optimising health outcomes within resource constraints.^28^

Despite these concerns, there are also compelling reasons to prioritise cancer R&D. Cancer is a leading cause of death globally and imposes a significant economic burden. The global cancer incidence rate is 440.5 per 100 000 people annually, and cancer-related deaths number 146.0 per 100 000 annually.^32^ In the USA, cancer care expenditures were $208.9 billion in 2020, with costs expected to rise as the population ages.^33^ Innovations in cancer therapies, such as immunotherapy and precision medicine, have already improved patient outcomes.^34 35^ Given cancer’s high mortality rate, prioritising research in this field is justified as an investment in saving lives. Furthermore, cancer R&D has historically driven medical innovation, benefiting other healthcare fields such as dementia and Alzheimer’s.^36^ Additionally, despite the high costs, cancer treatments have significantly improved survival rates and quality of life.^35^ For instance, the 5-year relative survival rate for breast cancer increased from 75% in the mid-1970s to 90% in 2011–2017.^37^ Innovations like immune checkpoint inhibitors have extended survival for previously untreatable cancers.^38^ Given the increasing global incidence of cancer, prioritising cancer R&D could lead to even more impactful treatments in the future. Public and political support for cancer research also plays a significant role in its prioritisation, as cancer is a cause that resonates deeply with the public.^39^ Furthermore, with 75% of global cancer deaths projected to occur in low-income and middle-income countries by 2030,^32^ prioritising cancer research can help address a global health crisis. Innovations in cancer treatment may also be adaptable to other diseases, further justifying a focus on oncology. In this context, prioritising cancer research is a strategic move to tackle one of the most pressing health challenges of the 21st century. So, while these arguments may support a somehow limited political emphasis on R&D efforts in cancer care, the question then remains: can onco-exceptionalism be justified?

## Can onco-exceptionalism be justified?

While cancer is undeniably a global health crisis with profound social and economic implications, the rationale for treating it as an exceptional case is fraught with oversimplifications that fail to capture the complexity of the disease and its treatment.

At the core of onco-exceptionalism is the promise of precision oncology, which aims to tailor treatment regimens based on individuals’ genetic and/or epigenetic profiles. This approach has indeed heralded substantial progress in some cancer types, offering new avenues for targeted therapies. However, the practical implications of precision oncology are constrained by the heterogeneous nature of cancer itself. Despite its transformative potential, the applicability of precision medicine remains limited to certain cancer types, and even within these, not all patients will benefit equally. As argued elsewhere,^34^ while precision medicine has revolutionised the treatment landscape for a subset of cancers, many others, particularly those diagnosed at later stages, lack actionable molecular markers or effective therapeutic targets.

A case in point is chimeric antigen receptor (CAR) T-cell therapy, which has shown remarkable success in haematologic cancers, but its benefits are not universally applicable. The exorbitant cost of this therapy—often exceeding $475 000 per treatment—raises significant concerns about its cost-effectiveness, particularly given that for many patients, the survival benefits are limited to less than 12 months.^31 36^ The search for universal ‘magic bullet’^40^ therapies for all cancer types has yet to be realised, and many cancers remain resistant to targeted therapies. This limitation suggests that while onco-exceptionalism may be appropriate in certain contexts, it risks oversimplifying the reality of cancer care by assuming that all patients will ultimately benefit from the same innovations. A more balanced approach would consider the specific needs of different cancer populations and the varying levels of benefit that precision medicine can provide.

The issue of cost-effectiveness further complicates the ethical debate around onco-exceptionalism. Many of the most advanced cancer treatments, particularly those involving precision medicine, fail to meet traditional cost-effectiveness thresholds, given their high costs and limited benefits.^41^ Treatments such as CAR T-cell therapy, while groundbreaking for some patients, are extremely expensive and offer only modest survival benefits for many others.^42 43^ Onco-exceptionalism, in this context, can exacerbate disparities in healthcare by directing substantial resources towards treatments that may not be cost-effective or universally beneficial.

The prioritisation of cancer care often assumes that any extension of life is inherently valuable. Yet, the pursuit of life extension through high-cost, high-risk interventions such as CAR T-cell therapy or other precision medicine treatments can sometimes result in significant physical suffering, emotional distress, social deprivation due to extended hospital stays, and diminished functional capacity.^44^ This highlights a critical ethical dilemma: can we justifiably place such a high premium on the extension of life without considering the broader implications for the patient’s overall quality of life? Moreover, in a world where healthcare systems are increasingly under strain, can it be ethically justified to allocate limited resources to treatments that do not deliver proportionate benefits in terms of survival or quality of life?

## From disease to health and its determinants—a broader framework for ethical deliberation

The ethical implications of onco-exceptionalism are highlighted when examined through alternative healthcare frameworks, such as Lennart Nordenfelt’s theory of health and the capabilities approach proposed by Amartya Sen and Martha Nussbaum.

Nordenfelt’s theory views health as more than the absence of disease—it emphasises the ability to function effectively in society and live a meaningful life.^45^ According to this framework, healthcare systems should not merely focus on curing disease but should also promote well-being and life satisfaction. From this idealistic perspective, onco-exceptionalism may be problematic because it often centres on the pursuit of curative cancer treatments, which, while important, may not always align with broader conceptions of health and well-being. Similarly, the capabilities approach provides a framework for evaluating healthcare that goes beyond merely extending life or curing disease. Health is seen as a core capability that enables individuals to pursue lives they value.^46^ This framework advocates for healthcare systems that prioritise prevention, equity and social determinants of health. It suggests that resources might be better allocated to initiatives promoting health across populations, reducing health disparities and addressing root causes of illness.

Moreover, the critique of the biomedical model by Venkatapuram adds another layer of complexity to the ethical considerations surrounding onco-exceptionalism.^47 48^ Venkatapuram argues that health outcomes are deeply influenced by social and environmental factors, such as lifestyle, socioeconomic status and access to healthcare, which are often overlooked in the biomedical model’s focus on disease treatment. For example, lung cancer, frequently linked to smoking, exemplifies how health outcomes are shaped by factors like personal behaviour and socioeconomic circumstances. Venkatapuram’s critique underscores that focusing solely on curative cancer treatments risks neglecting the broader social context that contributes to poor health in the first place.

## Discussion

The question of whether onco-exceptionalism can be justified leads to a multifaceted conclusion. On one hand, onco-exceptionalism advocates for prioritising cancer care due to the significant burden it places on global health systems, particularly in terms of resource allocation for innovative therapies. This argument is compelling, especially in the context of societal commitment to providing optimal oncological care. However, a closer examination reveals key challenges. Cancer is a heterogeneous group of diseases, each with varying treatment responses, survival rates, genetic and epigenetic complexities. This diversity weakens the justification for uniform prioritisation of oncology, as it fails to address the varied needs within the field. Furthermore, neither efficiency nor justice, central tenets of healthcare resource allocation, sufficiently supports the case for onco-exceptionalism on its own.^49^

Additionally, as pointed out by Krzyszczyk *et al*,^34^ precision medicine shows great promise in enhancing therapeutic outcomes by customising treatments according to the specific biology of individual patients. The Personalized Medicine Coalition^50^ also emphasises the potential of precision and personalised medicine to lower healthcare expenses for both patients and payers by categorising patients into more refined subgroups, thus increasing the chances of treatment success. This method can streamline drug development and its clinical application, ultimately improving cost-effectiveness. However, as highlighted by Krzyszczyk *et al*,^34^ the move towards targeted therapies via patient stratification could shrink the potential market size for pharmaceutical companies, leading to higher prices to offset the smaller patient base. This creates a challenge for the economic sustainability of precision medicine, especially in markets where there is limited flexibility in pricing and where negotiations between buyers and sellers influence the cost structure. Therefore, while precision medicine has the potential to transform cancer treatment, its economic viability and fair distribution of resources are still uncertain, with considerable consequences for healthcare budgets and patient access to care.

An alternative to onco-exceptionalism is to prioritise exceptional medical strategies that demonstrate broad population benefits, rather than focusing solely on one disease. This redefined form of exceptionalism would advocate for prioritising medical strategies that yield significant benefits across broader populations, rather than focusing on one disease. Precision medicine as mentioned above, for instance, offers potential benefits. As a standalone strategy, however, it may risk exacerbating the financial burden on healthcare systems, benefiting only a small subset of patients who experience positive outcomes. However, when integrated into a broader framework—such as 4P medicine (predictive, preventive, participatory and personalised care)—precision medicine could contribute to lowering overall disease burden and remain financially sustainable. In this paradigm, precision medicine would complement preventive approaches, ensuring that expensive interventions remain affordable by reducing the need for their widespread application. If precision medicine is conceptualised as part of an integrated health system that emphasises prevention, risk adaptation and active patient participation, its potential becomes more viable. In such a system, wellness promotion and preventive care would take precedence, leading to lower disease burdens and reduced reliance on costly medical interventions. This would allow for a more sustainable allocation of resources, making even expensive treatments more justifiable. However, realising this vision would require a profound rethinking of healthcare systems, including shifting financial priorities to foster healthier living at both the individual and societal levels. This transformation involves not only addressing the responsibilities of individuals but also recognising the shared obligations within society to promote public health.

The rising costs of cancer care underscore the need for a shift in healthcare policy, focusing on prevention, wellness and health equity. This shift involves investing in preventive care, such as early detection, lifestyle changes and addressing social determinants of health, supported by AI-driven decision-making to optimise resource allocation and care delivery. Preventive measures like smoking cessation and early cancer screenings reduce both disease burden and costs.^51^ Smoking cessation has been shown to significantly reduce lung cancer rates, while early detection methods like mammograms lower cancer mortality and treatment expenses.^52 53^ Lifestyle changes, including improved diet and exercise, prevent up to one-third of cancers.^54^ Programmes like Finland’s North Karelia Project demonstrate the effectiveness of behaviour change in reducing cancer rates and healthcare costs.^55^ Preventive care not only improves health but is also economically beneficial.

While this vision of a redefined healthcare system may seem utopian, its necessity has never been clearer. The financial pressures on healthcare systems, exacerbated by rising treatment costs and growing social inequalities, will soon force us to confront the limitations of current models. This growing inequity will make it increasingly difficult to justify the exceptional prioritisation of cancer treatment, particularly if such prioritisation comes at the expense of broader, more cost-effective health initiatives. A more pragmatic alternative, in response to these challenges, might be to devise decision-making machinery^56^ that embraces a deliberative process (akin to Deliberative Appraisal Processes in Health Technology Assessment^57^ and Stratil *et al*^58^) and flexible criteria. This approach would allow for a nuanced evaluation of exceptionalism based on the nature, seriousness and context of each intervention.

## Conclusion

In conclusion, while there is no dispute that precision medicine offers significant potential benefits for patients, particularly within oncology, this should not automatically warrant an exceptional allocation of resources towards its implementation. It is critical to expand the focus beyond treatment to include preventive strategies, which often receive far less attention. Onco-exceptionalism, as outlined in the manuscript, contributes to a framework that exceptionalises cancer R&D, pricing, reimbursement and approval processes. These exceptional practices in oncology, while designed to advance cancer care, can divert resources from other equally important areas of healthcare.

A more balanced approach to healthcare resource allocation—one that considers not only curative treatments but also prevention, social determinants of health and equitable access to care—may provide a more ethically sound framework for addressing the challenges of cancer treatment and healthcare allocation as a whole. By ensuring that healthcare resources are distributed in ways that promote well-being and address the root causes of illness, healthcare systems can better support all individuals in leading healthy, fulfilling lives.

## Data Availability

Data sharing not applicable as no datasets generated and/or analysed for this study.
